# Characteristics of clients who access mobile compared to clinic HIV counselling and testing services: a matched study from Cape Town, South Africa

**DOI:** 10.1186/s12913-014-0658-2

**Published:** 2014-12-20

**Authors:** Sue-Ann Meehan, Pren Naidoo, Mareli M Claassens, Carl Lombard, Nulda Beyers

**Affiliations:** Department of Paediatrics and Child Health, Desmond Tutu TB Centre, Faculty of Medicine and Health Sciences, Stellenbosch University, Francie van Zijl Ave, Parow, Cape Town South Africa; Biostatistics Unit, Medical Research Council, Francie van Zijl Ave, Parow, Cape Town South Africa

**Keywords:** HIV counselling and testing, HIV risk, Mobile HCT, Clinic HCT, Males, “Opportunistic” visits, Matched pairs

## Abstract

**Background:**

Studies within sub-Saharan African countries have shown that mobile services increase uptake of HIV counselling and testing (HCT) services when compared to clinics and are able to access different populations, but these have included provider-initiated HCT in clinics. This study aimed to compare the characteristics of clients who self-initiated HCT at either a mobile or a clinic service in terms of demographic and socio-economic variables, also comparing reasons for accessing a particular health service provider.

**Methods:**

This study took place in eight areas around Cape Town. A matched design was used with one mobile HCT service matched with one or more clinics (offering routine HCT services) within each of the eight areas. Adult clients who self-referred for an HIV test within a specified time period at either a mobile or clinic service were invited to participate in the study. Data were collected between February and April 2011 using a questionnaire. Summary statistics were calculated for each service type within a matched pair and differences of outcomes from pairs were used to calculate effect sizes and 95% confidence intervals.

**Results:**

1063 participants enrolled in the study with 511 from mobile and 552 from clinic HCT services. The proportion of males accessing mobile HCT significantly exceeded that of clinic HCT (p < 0.001). The mean age of participants attending mobile HCT was higher than clinic participants (p = 0.023). No significant difference was found for socio-economic variables between participants, with the exception of access to own piped water (p = 0.029). Participants who accessed mobile HCT were significantly more likely to report that they were just passing, deemed an “opportunistic” visit (p = 0.014). Participants who accessed clinics were significantly more likely to report the service being close to home or work (p = 0.035).

**Conclusions:**

An HCT strategy incorporating a mobile HCT service, has a definite role to play in reaching those population groups who do not typically access HCT services at a clinic, especially males and those who take advantage of the opportunity to test. Mobile HCT services can complement clinic services.

## Background

South Africa has an AIDS epidemic with primarily heterosexual transmission [1]. HIV prevalence in South Africa is 18% among the general adult population and 30% among pregnant women. The total number of people living with HIV continues to grow [1]. Within the Western Cape Province of South Africa, the estimated HIV prevalence in the age group 15–49 years has increased from 5.2% in 2008 to 9.2% in 2012 [2]. There are significant variations at district and sub-district level with possible contributing factors including poor levels of education, transactional sex, migration from areas of higher HIV prevalence, poverty and unemployment [3].

The government’s National Strategic Plan on HIV, STIs and TB (2012–2016) is a coordinated response to this epidemic, with the aim of reducing the number of new HIV infections by 50% and ensuring that at least 80% of people who are eligible for treatment for HIV are receiving it [4]. Knowing one’s HIV status is key to achieving these goals and is a key component of HIV care and prevention [5-7]. HCT also encourages safer sex and is an entry point for HIV care and treatment services

Demographic and socioeconomic factors have been associated with HIV risk. Unmarried men have been shown to be at highest risk for HIV followed by unmarried women [8]. Individuals who are divorced, separated or widowed tend to have higher HIV prevalence [9]. Educational attainment can be associated with lower risk of HIV infection [10]. Poverty and those living in informal settlements has been shown to be a social determinant for HIV infection [11,12].

Understanding the characteristics of clients who self-initiate for HCT can assist to identify if high risk individuals are accessing services. Studies have looked at different strategies of HCT within sub-Saharan Africa and have shown that mobile or home-based services, compared to clinics, increase uptake of HCT [13-15], are able to reach the largest proportion of previously untested people [16-18] including males [18-20], across age groups [18,19,21], working people [22] and less educated populations [21]. Mobile HCT was also deemed to be convenient, confidential and credible [23,24]. However these studies have included all categories of clients attending clinic HCT, including those referred for HCT by a health professional.

This study aimed to compare the characteristics of clients who self-initiated HCT at either a mobile or a clinic service in the Cape Town district of the Western Cape Province of South Africa. We wanted to determine if mobile services attracted a different population of clients. We also assessed the reasons for clients choosing the service.

## Methods

### Study areas

Community HCT centres were established in 2008 by the Desmond Tutu TB Centre (DTTC) at Stellenbosch University in partnership with non-governmental organizations (NGOs), in eight high HIV and TB burdened communities within the Cape Town health district of the Western Cape Province of South Africa. The settings varied with some centres in shopping malls and others in residential areas. Staff provided HCT either from the community HCT centres or on an outreach, mobile basis. The latter entailed services being provided from pop-up tents and a caravan (mobile van) in an appropriate open space, including taxi ranks or train stations, on open fields, next to main routes or close to public meeting places. Mobile services were offered during standard clinic operating hours during the study period.

During HCT, clients underwent pre-test counselling; received education, were symptomatically screened for TB and STIs, family planning needs were assessed and consent for an HIV test was taken. Both HIV and TB testing were conducted in accordance with national algorithms. A client was diagnosed with HIV if both the screening and confirmatory rapid tests were positive. Discordant results were confirmed with a laboratory ELISA HIV test. Clients received their HIV test result during post-test counselling. HIV positive clients were referred to the clinic of their choice for ongoing care. Trained HIV lay counsellors provided counselling and a nurse provided clinical services and HIV testing. Monitoring and evaluation activities, including a bi-annual audit, ensured standardised quality services.

The HCT process in clinics differed. The ACTS (advise, consent, test, support) model was used. Pre-test counselling is abbreviated, allowing counselling to be delivered within a few minutes to enable providers to integrate HIV testing into routine care. Issues specific to the client’s HIV status as well as aspects typically dealt with during pre-test counselling are addressed during post-test counselling. Clients who were diagnosed as HIV positive were referred to HIV services. Trained HIV lay counsellors provided both the counselling and the HIV testing.

The eight geographical areas in which the study took place are under-developed, densely populated peri-urban areas, with high unemployment rates and high TB and HIV disease burden.

### Design

A matched design, comparing mobile with clinic HCT services, within a geographically defined area, was utilised. A 2 km radius was plotted around each of the eight community HCT centres to circumscribe eight geographical areas. All clinics within these geographical area were included with between one and five clinics matched to each mobile service. There was no overlap between these eight areas. Only services within these areas were included in the study.

### Definitions

Mobile HCT: HIV counselling and testing services provided on an outreach basis by a local non-governmental organization.

Clinic HCT: HIV counselling and testing services provided within a primary health care clinic.

Self-initiated HCT: Individuals who actively seek HIV testing and counselling at a facility that offers these services [5].

Provider-initiated HCT: HIV testing and counselling that is recommended by health care providers [5].

### Study population

This study included clients who self-initiated (actively sought an HIV test) to either the mobile HCT service or one of the clinics within each of the geographically defined areas. Clients who received provider-initiated HCT at clinics were excluded. All adult clients (18 years of age or older), irrespective of their place of residence, were offered the opportunity to participate in the study. Clients were enrolled sequentially, after they had completed HCT, until the target number for each service and area was reached. The study could not differentiate between clients who refused participation and those who were not referred by HCT counsellors. These clients were identified through the routinely collected data.

### Sample size

To show a significant gender differential, we assumed 60% of participants being tested in mobile services are male versus 40% in the clinics, based on routine data. With a two sided confidence interval of 95% and power of 80%, the required total sample size was 960 for the eight areas. This equated to a total of 120 participants per area (60 from mobile and 60 from the clinics). Equal numbers of participants were enrolled from the clinics within an area. In two areas, with more than three clinics a minimum of twenty participants per clinic were enrolled.

### Methods

A questionnaire was developed to collected data on demographic and socio-economic variables, as well as reasons for accessing a particular health service provider. The questionnaire was translated from English into Afrikaans and isiXhosa and back translated into English by two different linguists and piloted in the same communities. The pilot data were not included in the study. Enrolment took place between February and April 2011. Written informed consent was obtained from each participant, with the option to leave the study at any point. The questionnaire was administered by trained research assistants at the site after completion of HCT.

### Analysis

Summary statistics were calculated for mobile and clinic HCT services within each geographical area. A matched pair consisted of all the clinics compared to the mobile service within a geographic area. A matched pair analysis was done, taking the differences within each pair and calculating the mean of the differences across pairs, to estimate the effect size and confidence interval. A permutation test was used to evaluate the significance of the differences between the two services (mobile or clinic).

### Ethics approval

The study was approved by the Health Research Ethics Committee of Stellenbosch University and The International Union against Tuberculosis and Lung Disease Ethics Advisory Group. Permission was obtained from the Cape Town City Health Directorate and the Western Cape Government Department of Health to undertake the research in their facilities.

## Results

A total of 1063 participants were enrolled in the study; 511 from mobile HCT services and 552 from clinic HCT services.

Routine data collected during the study period showed that 1049 clients self-initiated HCT at mobile services and 813 at clinic services. The study population is similar to the general population who self-initiated mobile and clinic HCT services in terms of sex (Table 1). Due to a large proportion of missing age variables for those who attended HCT services but did not participate in the study, we are not able to draw a similar comparison in terms of age.

**Table 1:** **A comparison of study participants and non-participants at mobile and clinic HCT services for sex and age**

	**Mobile HCT**			**Clinic HCT**		
	**Study data: participants n (%)**	**Routine data: non-participants n (%)**	**p -value**	**Study data: participants n (%)**	**Routine data: non-participants n (%)**	**p-value**
**Sex**	**n = 511**	**n = 538**		**n = 552**	**n = 261**	
Males	261 (51)	276 (51)	p = 0.942	149 (27)	69 (26)	p = 0.867
Females	250 (49)	262 (49)		403 (73)	192 (74)	
*Age						
18-25 yrs	158 (31)	117 (28)		228 (41)	20 (24)	
26-30 yrs	110 (22)	72 (17)		124 (23)	46 (56)	
31-50 yrs	201 (39)	192 (46)		179 (32)	12 (15)	
>50 yrs	42 (8)	38 (9)		21 (4)	4 (5)	
Missing	0	119		0	179	

*Age proportions are calculated on recorded values only.

In all 8 areas the proportion of males accessing mobile HCT exceeded that of clinic HCT. In total 261 (51%) were male in mobile compared to 149 (27%) in clinics (p < 0.001) (Table 2).

**Table 2:** **A comparison of demographic variables of participants at mobile and clinic HCT services**

	**Mobile n (%)**	**Clinic n (%)**	Effect size* % (95% CI)	**p value**
**Gender**	**n = 511**	**n = 552**		
Males	261 (51)	149 (27)	23.7 (12.1 to 35.3)	p < 0.001
Females	250 (49)	403 (73)		
**Age**	**n = 511**	**n = 552**		
Mean age	33 (sd = 11.29)	30 (sd = 9.49)	Years 2.8 (0.9 to 4.8)	p = 0.023
18-25 yrs	158 (31)	228 (41)		p < 0.001
26-30 yrs	110 (22)	124 (23)		p = 0.712
31-50 yrs	201 (39)	179 (32)		p = 0.019
>50 yrs	42 (8)	21 (4)		p = 0.002
**Marital status**	**n = 508**	**n = 552**		
Married	135 (27)	179 (32)	6.0 (−3.0 to 16.0)	p = 0.256
Single	373 (73)	373 (68)		
**Education**	**n = 510**	**n = 549**		
Incomplete or no schooling	305 (60)	331 (60)	1.0 (−9.0 to 12.0)	p = 0.769
Completed schooling	205 (40)	218 (40)		

*Effect size estimated for the first category of variable.

The mean age of participants attending mobile HCT was higher than clinic participants (Table 2). The proportion of clients, 31 to 50 years and older than 50 years, were significantly more likely to access mobile than clinic services. Neither the marital status (p = 0.256) nor educational attainment (p = 0.769) of participants who accessed mobile HCT differed significantly from those who accessed clinic HCT (Table 2). Of the total study population, 31% had completed schooling and a further 9% had tertiary education.

No significant difference was found for socio-economic variables between participants who accessed mobile and clinic services (Table 3) with the exception of access to own piped water, defined as piped water in their house or yard. Participants who accessed mobile HCT reported significantly less access to their own piped water (p = 0.029).

**Table 3:** **A comparison of socio-economic variables of participants at mobile and clinic HCT services**

	**Mobile n (%)**	**Clinic n (%)**	Effect size* % (95% CI)	**p value**
**Employment status**	**n = 509**	**n = 550**		
Formal	111 (22)	150 (27)	−5.7 (−3.2 to 14.6)	p = 0.172
Informal/unemployed	398 (78)	400 (73)		
**Social grant**	**n = 506**	**n = 551**		
No social grant	432 (85)	444 (81)	−5.0 (−10.0 to 9.0)	p = 0.453
Social grant	74 (15)	107 (19)		
**Housing type**	**n = 510**	**n = 550**		
Brick	244 (48)	276 (50)	−4.0 (−20.0 to 14.0)	p = 0.640
Informal	266 (52)	274 (50)		
**Drinking water**	**n = 511**	**n = 547**		
Own piped water	393 (77)	458 (84)	7.0 (−2.0 to 11.0)	p = 0.029
Public piped water	118 (23)	89 (16)		
**Fuel for cooking**	**n = 511**	**n = 551**		
Electricity	480 (94)	496 (90)	−4.0 (−8.0 to 1.0)	p = 0.072
Other fuel source	31 (6)	55 (10)		

*Effect size estimated for the first category of variable.

In both groups, the majority were unemployed, lived in informal housing, and had access to electricity for cooking.

The reasons for selecting the service provider differed (Table 4). Participants who accessed mobile HCT services were significantly more likely to be passing by and took advantage of the opportunity to test (p = 0.014). Participants who accessed clinic HCT services were significantly more likely to access the clinic because it was in close proximity to home or work (p = 0.035). Perceptions of short queues and quick service did not influence the choice of service provider.

**Table 4:** **A comparison of reasons for selecting a service provider**

		**Mobile n (%)**	**Clinic n (%)**	Effect size* % (95% CI)	**p value**
		**n = 510**	**n = 548**		
Passing by (opportunistic)	yes	246 (48)	6 (1)	47.0 (27.0 to 67.0)	p = 0.014
Passing by (opportunistic)	no	264 (52)	542 (99)		
Close to home/work	yes	213 (42)	399 (73)	−31.0 (−51.0 to 31.0)	p = 0.035
Close to home/work	no	297 (58)	149 (27)		
Short queues	yes	69 (14)	13 (2)	10.8 (−12.1 to 33.7)	p = 0.353
Short queues	no	441 (86)	535 (98)		
Quick service	yes	109 (21)	64 (12)	0.9 (−10.0 to 29.0)	p = 0.358
Quick service	no	401 (79)	484 (88)		

*Effect size estimated for the first category of variable.

No significant difference regarding travel time or travel cost was found between participants accessing either service (Table 5). The majority of participants (91%) spent less than 30 minutes travelling to the service. Overall 88% incurred no out of pocket travel cost in accessing the service. The majority of those who incurred a cost paid less than US$ 2 (exchange rate US$ 1: ZAR 10)

**Table 5:** **A comparison of travel and waiting times of participants at mobile and clinic HCT services**

	**Mobile n (%)**	**Clinic n (%)**	Effect size* % (95% CI)	**p value**
**Travel time**	**n = 506**	**n = 550**		
≤30 min	462 (91)	505 (92)	0 (−8.0 to 8.0)	p = 0.872
>30 min	44 (9)	45 (8)		
**Travel cost**	**n = 434**	**n = 462**		
No cost	397 (91)	395 (85)	−2.0 (−5.0 to 1.0)	p = 0.119
Cost incurred	37 (9)	67 (15)		
**Waiting time**	**n = 509**	**n = 552**		
<15 min	422 (83)	214 (39)	43.0 (15.0 to 69.0)	p = 0.030
≥15 min	87 (17)	337 (61)		
**Total time**	**n = 510**	**n = 552**		
<30 min	447 (88)	408 (74)	14.0 (9.0 to 30.0)	p ≤ 0.001
≥30 min	63 (12)	144 (26)		

*Effect size estimated for the first category of variable.

Waiting times were significantly shorter for clients accessing mobile services (p = 0.030). The maximum waiting time at mobile HCT services was 30 minutes and at clinic HCT services was 60 minutes. Although total time taken to go through counselling and testing was significantly shorter for clients accessing mobile services (p ≤ 0.001), the majority of clients, irrespective of where they accessed HCT services, took less than 30 minutes.

## Discussion

HIV risk is influenced by a variety of factors, including sex, age, educational status, marital status and poverty. HIV prevention and care is dependent on HCT services being accessible to those at risk.

In Cape Town, HCT services have been provided at clinics and through mobile services. The majority of those tested at clinics receive a provider-initiated service, based on clinical indications. Clinics also provide a range of services not provided by mobile HCT and therefore have the ability to attract a large proportion of clients. However, their HCT coverage is limited to populations groups who access clinics. All clients who access mobile services self-initiate. Routine data (2013) from the City of Cape Town clinics shows that 28% of clients self-initiate.

Studies conducted in Cape Town have shown that mobile HCT is a way to target other groups and is able to target more males and across age groups [20,25,26] compared to other HCT strategies. These studies have included provider-initiated HCT within clinics. This study is different as it includes self-initiated clients at both services and the study population were thus more comparable.

This study showed that significantly more males were counselled and tested through mobile HCT compared to clinic HCT within the study as a whole and within each of the matched pairs. This finding confirms the results of other studies conducted within the Cape Town district [25,27] and a study conducted in a rural area of another South African province [18]. It highlights the ability of mobile HCT to increase access to HCT for males, who traditionally do not access HCT at clinics. Flexibility of services allows mobile HCT to be provided at places where males typically congregate, like transport hubs (train stations and taxi ranks).

Overall, participants accessing mobile HCT were 3 years older than those accessing clinic HCT. Participants accessing mobile HCT were significantly more likely to be in the older age categories of 31–50 years and >50 years, while those accessing clinic HCT were significantly more likely to be in the 18–25 year age category. Younger people may access HCT at a clinic facility during visits for family planning or when taking their children for immunizations, making it easy to request an HIV test at the same time. Since this study was concluded, family planning services have been incorporated into Community-based HCT and we have seen a large increase in the number of young women accessing services. We have also considered that mobile HCT may not appeal to the youth and making the service more youth friendly may be a worthwhile opportunity. Earlier studies have shown that mobile HCT services can access both older age groups, [25] and youth [18,28]. More research is needed.

HIV prevalence in South Africa is much higher among young women compared to young men. Overall in age groups 15–19 years and 20–24 years, significantly more females are HIV-infected than their same age male peers [1,29]. HIV prevalence disparities by gender and age may be an indication of intergenerational sex [30]. HCT strategies that access older men and younger women play an important role in HIV prevention and care.

Overall, the socio-economic status of the two populations were similar, both were vulnerable in terms of education, levels of poverty and unemployment. Data from the South African Census (2011) for these communities showed that the majority of people had not completed school, were unemployed, lived in formal dwellings and used electricity for cooking [31] and were comparable to the study population.

Participants accessing either mobile or clinic HCT services were potentially living within these same impoverished areas. The only significant difference found in the study between the two populations was that clients who attended mobile HCT were significantly less likely to have access to their own piped drinking water.

Overall, the majority of participants were not formally employed. People not engaged in formal work are more likely to be in the community during the day. Mobile services have the opportunity to reach these people. The most common reason for working people not accessing HCT is not being able to leave work [22,28]. This provides an opportunity for mobile services to access work places directly. Being able to take services to a targeted population, thereby making the services accessible is a benefit of mobile services.

Mobile HCT was able to access a significantly higher proportion of participants, who were just passing by the service and were able to take advantage of the opportunity presented to them. We refer to these as “opportunistic” visits, as clients would otherwise not have accessed HCT at that time. This finding underlines the importance of taking the services to the people, in that people will utilize an opportunity to test and are willing to test if services are easily accessible. A significantly higher proportion of participants who accessed clinic HCT reported that the clinic was close to their home or work. This again highlights the importance of proximity in access. A study in Uganda showed the strongest predictors of satisfaction with services included accessibility, convenience and availability of services [32].

A significantly higher proportion of participants who accessed mobile HCT reported shorter waiting times before seeing a counselor. As longer waiting times are associated with higher levels of dissatisfaction with services [33], waiting time should be a consideration for clients who only want to access HCT. The majority of participants did not incur any travel costs and reported a travel time of less than thirty minutes. Costs in terms of time and money associated with accessing services as well as longer travelling distances and waiting times have been shown to have a negative association with use of services [34]. The ability of mobile services to reduce travel time and costs due to the high proportion of “opportunistic “visits, together with short waiting times, contributes to making HCT easily accessible and affordable .

The study had limitations. The communities were not randomly selected, but based on the sites in which mobile services were being implemented. The study was conducted within a limited geographical urban/peri-urban area. Any generalizability of results should be made with caution. Additional research needs to be carried out, including in rural and semi-rural areas.

We were unable to record HCT clients who were not referred to us or who refused participation, but the routine data collected for the same time period has been used and the uptake of participants is known and these were representative in terms of gender. We cannot be certain about the representativeness of the age data due to missing variables.

A major strength of the study is that the findings have provided detailed demographic and socio-economic data on the participants who self-initiated at mobile and clinic HCT services. This has allowed us to gain some insight into the characteristics of these populations and their potential risk for HIV based on their characteristics and impoverished circumstances.

The study has highlighted the strengths of mobile HCT services with the ability to target populations directly and thereby provide access to those who would not typically access services e.g. males and “opportunistic” clients. Short travelling times, little travel costs and short waiting times also make mobile services easily accessible. The study also identified that proportionately fewer youth attended mobile services and this provides the opportunity to deliver more youth friendly services.

Future research should determine the drivers and barriers to HCT at either mobile or clinic services through community surveys to assess those who have never tested previously and may be at high risk.

## Conclusions

Mobile services can access at risk populations, have the ability to increase access to males and those who are just passing and would otherwise not have accessed HCT at that time. Mobile HCT services provide the opportunity to target specific populations e.g. at workplaces, at transport hubs and within communities. The flexibility of mobile services can also allow access to populations that are currently being missed. Strategically, mobile HCT services should run in conjunction with clinic HCT services to reach all age groups and both sexes. If universal access to HCT is to be obtained, then mobile HCT services should be considered in the overall HCT strategy.

## Abbreviations

HCT: HIV counselling and testing
ELISA: Enzyme-linked immunosorbent assay

## References

[1] Fraser-Hurt N, Zuma K, Njuho P, Chikwava F, Slaymaker E, Hosegood V: *The HIV Epidemic in South Africa: What Do We Know and How Has It Changed?* South African National AIDS Council: South Africa; 2011.

[2] Shisana O, Rehle T, Simbayi LC, Zuma K, Jooste S, Zungu N, Labadarios D, Onoya D, Davids A, Ranlagan S, Mbelle M, van Zyl J, Wabiri N: *South African National HIV Prevalence, Incidence and Behaviour Survey.* Human Sciences Research Council HSRC Press: South Africa; 2014.

[3] Western Cape Provincial Government: *Provincial Strategic Plan on HIV/AIDS, STIs and TB 2012–2016*. National department of health: South Africa; 2012.

[4] National Department of Health: *National Strategic Plan on HIV, STIs and TB 2012–2016*. National department of health: South Africa; 2011.

[5] National Department of Health: *Policy Guideline for HIV Counselling and Testing (HCT)*. National department of health: South Africa; 2010.

[6] World Health Organization: *New Approaches to HIV Testing and Counselling care and preventing further infection*. National department of health: Geneva; 2003.

[7] Kalichman SC, Simbayi LC (2003). HIV testing attitudes, AIDS stigma, and voluntary HIV counselling and testing in a black township in Cape Town, South Africa. Sex Transm Infect.

[8] Bassett IV, Giddy J, Wang B, Lu Z, Losina E, Freedberg KA (2008). Routine, voluntary HIV testing in Durban, South Africa: correlates of HIV infection. HIV Med.

[9] United Nations Programme on HIV/AIDS (UNAIDS) and the World Health Organization (WHO): *AIDS Epidemic Update.* World health Organization: Switzerland; November 2009.

[10] Jukes M, Simmons S, Bundy D (2008). Education and vulnerability: the role of schools in protecting young women and girls from HIV in southern Africa. AIDS.

[11] Shisana O, Rice K, Zungu N, Zuma K (2010). Gender and Poverty in South in the era of HIV/AIDS: a quantitative study. J Womens Heal.

[12] Harrison D: *An Overview of Health and Health Care in South Africa 1994 – 2010: Priorities, Progress and Prospects for New Gains. A discussion document.* South Africa: 2010. [ftp.bhfglobal.com/files/bhf/overview1994-2010]

[13] Bateganya M, Abdulwadud O, Kiene S: **Home-based HIV voluntary counselling and testing (VCT) for improving uptake of HIV testing (Review).** *Cochrane Database of Systematic Review* 2010, issue 7. Art. No.: CD006493. doi:10.1002/14651858.CD006493.pub4. 10.1002/14651858.CD006493.pub4 PMC6464814 20614446

[14] Helleringer S, Hans-Peter K, Jemima F, James M (2009). Increasing uptake of HIV testing and counseling among the poorest in Sub-Saharan Countries through home-based service provision. J Acquir Immune Defic Syndr.

[15] Khumalo-Sakutukwa G, Morin SF, Fritz K, Charlebois ED, Van Rooyen H, Chingono A, Modiba P, Mrumbi K, Visrutaratna S (2008). Project accept (HPTN 043): a community-based intervention to reduce HIV incidence in populations at risk for HIV in Sub-Saharan Africa and Thailand. J Acquir Immune Defic Syndr.

[16] Peltzer K, Matseke G, Mzolo T, Majaja M (2009). Determinants of knowledge of HIV status in South Africa: results from a population-based HIV survey. BMC Public Health.

[17] Menzies N, Abang B, Wanyenze R, Nuwaha F, Mugisha B, Coutinho A, Bunnell R, Mermin J, Blandford JM (2009). The costs and effectiveness of four HIV counseling and testing strategies in Uganda. AIDS.

[18] Maheswaran H, Thulare H, Stanistreet D, Hons BA, Tanser F (2012). Starting a home and mobile HIV testing service in a rural area of South Africa. Test.

[19] Mutale W, Michelo C, Jürgensen M, Fylkesnes K (2010). Home-based voluntary HIV counselling and testing found highly acceptable and to reduce inequalities. BMC Public Health.

[20] Kranzer K, van Schaik N, Karmue U, Middelkoop K, Sebastian E, Lawn SD, Wood R, Bekker L-G: **High Prevalence of Self-Reported Undiagnosed HIV despite High Coverage of HIV Testing: A Cross-Sectional Population Based Sero-Survey in South Africa.** *PLoS One* 2011, **6**(9):e25244. doi:10.1371/journal.pone.0025244. 10.1371/journal.pone.0025244 PMC3182182 21969875

[21] Hood JE, MacKellar D, Spaulding A, Nelson R, Mosiakgabo B, Sikwa B, Puso I, Raats J, Loeto P, Alwano MG, Monyatsi B (2012). Client characteristics and gender-specific correlates of testing HIV positive: a comparison of standalone center versus mobile outreach HIV testing and counseling in Botswana. AIDS Behav.

[22] Ostermann J, Reddy E , Shorter MM, Muiruri C, Mtalo A, Itemba DK, Njau B, Bartlett J, Crump J, Thielman NM: **Who tests, who doesn’t, and why? Uptake of mobile HIV counseling and testing in the Kilimanjaro Region of Tanzania.** *PLoS One* 2011, **6**(1):e16488. doi:10.1371/journal.pone0016488. 10.1371/journal.pone.0016488 PMC3031571 21304973

[23] Angotti N, Bula A, Gaydosh L, Kimchi EZ, Thornton R, Yeatman S (2009). Increasing the acceptability of HIV counseling and testing with three C’s: Convenience, confidentiality and credibility. Soc Sci.

[24] Jurgensen M, Tuba M, Fylkesnes K, Blystad A (2012). The burden of knowing: balancing benefits and barriers in HIV testing decisions. A qualitative study from Zambia. BMC Health Serv Res.

[25] Van Schaik N, Kranzer K, Wood R, Bekker L-G (2010). Earlier HIV diagnosis – are mobile services the answer?. October.

[26] Nglazi MD, van Schaik N, Kranzer K, Lawn SD, Wood R, Bekker L-G: **An Incentivized HIV Counseling and Testing Program Targeting Hard-to-Reach Unemployed Men in Cape Town, South Africa.** *J Acquir Immune Defic Syndr* 2012, **59:**e28-e24. 10.1097/QAI.0b013e31824445f0 PMC3801093 22173039

[27] Western Cape Provincial Government: *Final Draft - Know Your Response Report.* National department of health: South Africa; 2010.

[28] Corbett EL, Dauya E, Matambo R, Cheung YB, Makamure B, Bassett MT, Chandiwana S, Munyati S, Mason PR, Butterworth AE, Godfrey-Faussett P, Hayes RJ: **Uptake of workplace HIV counselling and testing: a cluster-randomised trial in Zimbabwe.** *PLoS Med* 2006, **3**(7):e238. doi:10.1371/journal.pmed.0030238. 10.1371/journal.pmed.0030238 PMC1483908 16796402

[29] Rehle TM, Hallett TB, Shisana O, Pillay-van Wyk V, Zuma K, Carrara H, Jooste S: **A decline in new HIV infections in South Africa: estimating HIV incidence from three national HIV surveys in 2002, 2005 and 2008.** *PLoS One* 2010, **5**(6):e11094. 10.1371/journal.pone.0011094 PMC2885415 20559425

[30] Miles O, Barnighausen T, Tanser F, Lurie M, Newell ML (2011). Age-gaps in sexual partnerships: seeing beyond “sugar daddies.”. AIDS.

[31] Statistics South Africa: *Census 2011; Interactive data in SuperCROSS.* Pretoria; 2011. Retrieved from http://interactive.statssa.gov.za/superweb/login.do

[32] Nabbuye-Sekandi J, Makumbi FE, Kasangaki A, Kizza IB, Tugumisirize J, Nshimye E, Mbabali S, Peters DH (2011). Patient satisfaction with services in outpatient clinics at Mulago hospital, Uganda. Int J Qual Health Care.

[33] Wouters E, Heunis C, van Rensburg D, Meulemans H (2008). Patient satisfaction with antiretroviral services at primary health-care facilities in the Free State, South Africa–a two-year study using four waves of cross-sectional data. BMC Health Serv Res.

[34] Mubyazi GM, Bloch P, Magnussen P, Olsen ØE, Byskov J, Hansen KS, Bygbjerg IC (2010). Women’s experiences and views about costs of seeking malaria chemoprevention and other antenatal services: a qualitative study from two districts in rural Tanzania. Malar J.

